# Antiulcer Effect of *Senna multiglandulosa* via Increased Secretion of Mucus and Nonprotein Sulfhydryl Groups in an Experimental Murine Model

**DOI:** 10.1155/2022/7570294

**Published:** 2022-08-12

**Authors:** Miriam Palomino-Pacheco, Juan Pedro Rojas-Armas, Jorge Luis Arroyo-Acevedo, José Manuel Ortiz-Sánchez, Hugo Jesús Justil-Guerrero, Edwin César Cieza-Macedo, Norma Ramos-Cevallos, Mohammed Merae Alshahrani, Shafi Mahmud, Oscar Herrera-Calderon

**Affiliations:** ^1^Laboratory of Biochemistry, Faculty of Medicine, Universidad Nacional Mayor de San Marcos, Av. Miguel Grau 755, Lima 15001, Peru; ^2^Laboratory of Pharmacology, Faculty of Medicine, Universidad Nacional Mayor de San Marcos, Av. Miguel Grau 755, Lima 15001, Peru; ^3^Laboratory of Physiology, Faculty of Medicine, Universidad Nacional Mayor de San Marcos, Av. Miguel Grau 755, Lima 15001, Peru; ^4^Research Institute for Pharmaceutical Sciences and Natural Resources, Faculty of Pharmacy and Biochemistry, Universidad Nacional Mayor de San Marcos, Jr. Puno 1002, Lima 15001, Peru; ^5^Department of Clinical Laboratory Sciences, Faculty of Applied Medical Sciences, Najran University, 1988, Najran 61441, Saudi Arabia; ^6^Division of Genome Sciences and Cancer, The John Curtin School of Medical Research, The Shine-Dalgarno Centre for RNA Innovation, The Australian National University, Canberra, ACT 2601, Australia; ^7^Department of Pharmacology, Bromatology and Toxicology, Faculty of Pharmacy and Biochemistry, Universidad Nacional Mayor de San Marcos, Jr. Puno 1002, Lima 15001, Peru

## Abstract

Peptic ulcer is a universal condition that is a public health problem due to its prevalence, risk of complications and socioeconomic impact. This study aimed to determine the antiulcer effect of the hydroalcoholic extract from *Senna multiglandulosa* leaves against ethanol-induced gastric ulcer in rats. Thirty-six male albino Holtzman rats were assigned to six groups. Group I received physiological saline (PS) at doses of 10 mL/kg; group II: ethanol (PS + ethanol 5 mL/kg); group III; omeprazole 100 mg/kg/day (gold standard); groups IV, V and VI received doses of 100, 250 and 500 mg/kg/day of *S. multiglandulosa* extract, respectively. The stomach was removed to determine the ulcerative lesions and two sections of the glandular zone to carry out the analysis of the gastric mucus and sulfhydryl groups content. As result, *S. multiglandulosa* at doses of 250 and 500 mg/kg produced a significant decrease of the injured area, with values of 46.28 ± 7.95 mm^2^ and 6.91 ± 2.48 mm^2^, respectively (*P* < 0.001). The protective effect was showed at dose of 500 mg/kg (92.27%) and a significant increase in the production of mucus with a value of 83.13 ± 13.09 mg/mL/g of tissue (61.14%). The production of nonprotein sulfhydryl groups (NP-SG) also increased significantly at the three evaluated doses, being 250.34 ± 21.16 *μ*g/g tissue at dose of 500 mg/kg (119.94%). It is concluded that *S. multiglandulosa* extract protected against ethanol-induced gastric ulcer due to increased gastric mucus secretion and its antioxidant activity due to the generation of nonprotein sulfhydryl groups.

## 1. Introduction

The concept of peptic ulcer disease (PUD) is named to an acid-induced lesion of the stomach and duodenum which are the areas commonly affected. In addition is characterized by a denuded mucosa, extending into the submucosa or muscularis propria [1, 2]. The pathophysiology is explained as a consequence in the imbalance between defense factors (mucin, bicarbonate, prostaglandin, nitric oxide and others) and damaging factors such as acid and pepsin. Within the main causes of PUD is the chronic use of drugs such as those belong to nonsteroidal anti-inflammatory drug (NSAID) family, mainly acetaminophen, naproxen, diclofenac and others. Otherwise, the infection by *Helicobacter pylori* is also linked to ulcer gastric and gastric cancer [3]. According to Xie et al. [4], the global prevalence of PUD was approximately 8.09 million in 2019, which represents 25.82% more than in 1990. Furthermore, the south Asia region had the highest age-standardized prevalence rate in PUD with 156.62 (130.58–187.05) per 100,000 population.

The study of experimental models to evaluate the bioactive metabolite or constituent chemicals of plants are focused on simulate the PUD in humans. Therefore, the most frequently used methods are NSAIDs and stress-induced model and pyloric ligation [5]. However, another model is based in the oral administration of ethanol in fasting rats, which is seemed to an acute human peptic ulcer condition. The main mechanism of acute damage is the destruction of the mucosal layer and exposes the mucosa to hydrochloric acid and pepsin. Additionally, microvascular injuries are induced by the ethanol leading to reduce the blood flow, increasing free radicals mainly reactive oxygenated species and proinflammatory chemicals such as cytokines [6].

Natural resources have been used since ancestral time as a source of products for the alternative treatment of various diseases. *Senna multiglandulosa* (Family: Fabaceae), known by the common name of millhua, mutuy, chanchayllo, among other. This species is distributed in Mexico, Colombia, Ecuador, Peru and Bolivia [7, 8]. In Peru, it is extended from Cajamarca to Puno between 2,300 and 4,000 meters above sea level [9, 10]. In traditional Peruvian medicine, *S. multiglandulosa* leaves are used in the treatment of labor pains and accelerate labor, gallbladder colic, kidney stones, deflate the blow and calm the pain; also, to fight cancer [11]. In addition, the ethnopharmacological information refers to the use of this plant for the treatment of gastric ulcer [12], but there are no scientific studies that validate this popular knowledge, so we carried out this research in order to evaluate the gastroprotective properties of the hydroalcoholic extract of *S. multiglandulosa* in a murine model.

## 2. Materials and Methods

### 2.1. Sample Collection

The fresh leaves of *Senna multiglandulosa* were collected in the province of Huancayo in the department of Junín, Peru (3,256 masl; latitude: −12.068065, longitude: −75.210553). A voucher of the plant was sent to the Herbarium of the Natural History Museum at the Universidad Nacional Mayor de San Marcos for taxonomic identification (Id. 86-USM-2016).

### 2.2. Extraction Process

The hydroalcoholic extract was prepared as follows: The leaves were washed with tap water, dried at 40°C, pulverized in a blade mill and then macerated in 80% ethanol for 7 days at room temperature with manually rotating for 15 minutes daily. Then, the solution was filtered using filter paper and a vacuum extraction pump, the filtrate was concentrated in a rotary evaporator and then placed in an oven at 40°C to remove the solvent. The dry residue (hydroalcoholic extract) was stored in an amber bottle at a temperature of 4°C until use.

### 2.3. Preliminary Phytochemical Study

For the preliminary phytochemical analysis of the hydroalcoholic extract of *Senna multiglandulosa*, different qualitative tests of secondary metabolites were carried out [13]:

- Molisch reaction to identify Carbohydrates: 0.5 mL of extract was taken and 0.15 mL of Molisch A reagent (2% alpha naphthol/ethanol) were added, homogenized and concentrated H_2_SO_4_was added, the reaction is positive by the formation of a purplish-red ring. .
- Ninhydrin reaction to identify Proteins: 0.5 mL of extract was taken and 0.15 mL of ninhydrin reagent (1% alpha naphthol/ethanol) were added, homogenized and placed in a water bath, the reaction is positive due to the formation of a purple chromogen.
- FeCl_3_ reaction to identify Phenolic Compounds: 0.5 mL of extract was taken and 0.15 mL of ferric chloride were added, it was homogenized and the reaction is positive due to the appearance of a bluish-green color.
- Collagen reaction to identify Tannins: 0.5 mL of extract was taken and 1 mL of collagen reagent (1 g%/10% NaCl) was added, the reaction is positive due to the appearance of a whitish color, indicating the presence of tannins
- Shinoda reaction to identify Flavonoids, Chalcones and Aurones: 0.5 mL of extract was taken and Magnesium and concentrated HCL were added 0.05 mL cada ve, the appearance of red color shows positive reaction and presence of flavonoids.
- Lieberman-Buchard reaction to identify steroids: The reaction takes place in zone, 0.5 mL of extract was taken and the Lieberman-Bouchard at reagent was added (CHCl_3_ acetic anhydride/concentrated H_2_SO_4_), the formation of green, blue, red or orange colors in the area indicates the positive presence of triterpenoids or steroids.
- Dragendorff reaction to identify alkaloids: 1 mL of extract was taken and 0.25 mL of Dragendorff reagent (potassium tetraiodine bismutate/acid) were added, homogenized, the reaction is positive due to the formation of a red-orange precipitate, indicating the presence of alkaloids.
- Mayer's reaction to identify alkaloids: 0.5 mL of sample was taken and 0.5 mL of Mayer's reagent (potassium tetraiodine mercurate) was added; the appearance of turbidity indicates a positive reaction for alkaloids.
- Fehling's reaction to identify Reducing Sugars: 1 mL of extract was taken and 0.5 mL of Fehling's reagent was added (CuSO_4_. 5 H_2_O and double sodium and potassium tartarate, Fehling A and B respectively) then it was placed in a water bath, the presence of a brick red precipitate shows a positive reaction for reducing sugars.
- Rosenheim reaction to identify Anthocyanins: 0.5 mL of extract was taken and 0.5 mL of Rosenheim's reagent (iodinated iodine solution) was added. The presence of a dark red color indicates the positive presence of catechin, flavonoids and anthocyanins.
- Foam reaction to identify saponins: 1 mL of sample was taken and 10 mL of water was added, vigorously stirred for one minute, the formation of foam with a height of 0.5 to 1 cm for a time of 15 minutes evidences the presence saponin positive.

### 2.4. Determination of Total Phenols Content (TPC)

The total phenols content was evaluated using the Folin-Ciocalteu method [14]. Different concentrations of *S. multiglandulosa* extract (500 *μ*L of each concentration) were mixed with Folin-Ciocalteu reagent (500 *μ*L) and deionized water (2500 *μ*L). The reaction was carried out for 10 min and then was added 1500 *μ*L of 20% (w/v) sodium carbonate. After 30 min, the absorbance was read at 760 nm using a Thermo Scientific Genesys 10S UV-VIS spectrophotometer. The TPC was calculated using the gallic acid calibration curve within the range of 10–100 *μ*g/mL (*R*^2^ = 0.9983). The results were expressed as mg of gallic acid equivalents (GAE)/g of extract.

### 2.5. Determination of Total Flavonoid Content

Total flavonoid content was determined using a colorimetric method described by Dewanto et al. [15]. Different concentrations of *S. multiglandulosa* (0.25 mL) was mixed with 1.25 mL of distilled water and 75 *μ*L of 5% NaNO_2_ solution to react for 6 min. Then, 150 *μ*L of a 10% AlCl_3_‚ 6H_2_O solution was added to the working solution and reacted for 5 min before adding 0.5 mL of 1 M NaOH. Then, distilled water was added to complete 2.5 mL of volume for each tube. The absorbance was measured immediately against a blank at 510 nm using a Thermo Scientific Genesys 10S UV-VIS spectrophotometer. The results were expressed as equivalent to catechin per gram of extract.

### 2.6. Determination of Antioxidant Capacity: Reducing Power Test

The reducing power of Fe^3+^ of the hydroalcoholic extract of the leaves of *S. multiglandulosa* was determined according to the method of Herrera-Calderon et al. [16]. All tests were performed in triplicate and the graph was plotted with the average of three observations.

### 2.7. Evaluation of the Gastroprotective Effect

#### 2.7.1. Effect on Ethanol-Induced Gastric Ulcers

The experiment was performed according to the method described by Zakaria et al. [17] with slight modifications. Male Holtzman albino rats were fasted for 24 hours before the study. Thirty-six rats, with a body weight of 220 ± 10 g, were randomly assigned to 6 groups as follows:

- Group I received Physiological saline (PS) at 10 mL/kg (negative control);
- Group II, PS + ethanol (positive control);
- Group III, omeprazole 100 mg/kg/day (gold standard);
- Groups IV, V and VI, at doses of 100, 250 and 500 mg/kg/day of the hydroalcoholic extract of *S. multiglandulosa*, respectively.

After 1 hour of treatment, ulceration gastric was induced to groups II, III, IV, V, and VI by oral administration of absolute ethanol (5 mL/kg). Then, animals were anesthetized using ether and sacrificed by thiopental (100 mg/Kg, via intraperitoneal) 1 h after ulcer induction. An abdominal laparotomy was performed and stomachs were removed to the histological analysis and macroscopic evaluation. Hemorrhagic and ulcerative lesions were measured using Image J computer software, v.153 g (NIH, USA). Ulcerated area (UA) was measured in mm^2^. The protection percentage was calculated using the following formula:

(1) $$\begin{array}{c} \%\text{Protection }=\frac{\left({\text{UA}}_{\text{vehicle}}-{\text{UA}}_{\text{extract}}\right)}{\left({\text{UA}}_{\text{vehicle}}\right)}\times 100, \end{array}$$

where UA vehicle, ulcerated area of vehicle; UA, ulcerated area of extract.

#### 2.7.2. Determination of Gastric Mucus and Nonprotein Sulfhydryl Groups

Two sections of the glandular zone of each stomach was used for analysis of the gastric mucus and sulfhydryl groups content. Gastric mucus production was determined by Ahmed modified spectrophotometric method [18], based on the adsorption of Alcian blue by gastric mucus, which was then extracted with a MgCl_2_ solution, and subsequently read at 598 nm. A calibration curve of Alcian blue, with concentrations from 3.0 to 50.0 *μ*g/mL, was prepared. The results were expressed in µg Alcian blue/mL/g of tissue and as a percentage of increase in gastric mucus, according to the following formula:

(2) $$\begin{array}{c} \%\text{increase inmucus}=\frac{\left({\text{GMI}}_{t}-{\text{GMI}}_{c}\right)}{{\text{GMI}}_{c}}\times 100. \end{array}$$

Where GMI_*t*_, gastric mucus index of the treatment GMI_*c*_, gastric mucus index of the control.

The determination of nonprotein sulfhydryl groups (NP-SG) was determined by the method of Sedlak and Lindsay [19], having as principle the recognition of sulfhydryl groups by 5,5′-dithiobis (2-nitrobenzoic) acid, which was read at 412 nm, in a spectrophotometer. To determine the concentration of GS-NP, a calibration curve was prepared using glutathione (GSH) as a standard, ranging from 3.0 to 50.0 *μ*g/mL The results were expressed in *μ*g of NP-SG/mL/g of tissue and in percentage increase of NP-SG, using the following formula:

(3) $$\begin{array}{c} \%\text{ increase in NP}-\text{SG}=\frac{\left({\text{NPSGI}}_{t}-{\text{NPSGI}}_{c}\right)}{{\text{NPSGI}}_{c}}\times 100. \end{array}$$

Where, NPSGI_*t*_, nonprotein sulfhydryl group index of the treatment, NPSGI_*c*_, nonprotein sulfhydryl group index of the control.

### 2.8. Statistical Analysis

Each evaluation in the antiulcerative effect were expressed as the mean ± SD and analyzed by one-way analysis of variance (ANOVA) followed by Tukey's multiple comparisons test using SPSS ver. 24.0 program (IBM Corporation, USA). *P* < 0.05 is considered as significant in each evaluated test.

### 2.9. Ethical Considerations

This protocol was approved by the ethics committee of the Faculty of Medicine (Id. 0287; May 20, 2019) of the Universidad Nacional Mayor de San Marcos (Lima, Peru).

## 3. Results

### 3.1. Phytochemical Analysis

The percentage of obtained extract was 11.25%. In the hydroalcoholic extract of *S. multiglandulosa* several groups of secondary metabolites were identified according to the preliminary screening. Phenolic compounds were found in higher concentration, followed by alkaloids and steroids, as shown in Table 1.

### 3.2. Antioxidant Activity

The total phenolic content of *S. multiglandulosa* was 10.51 ± 0.40 mg of gallic acid equivalents (GAE)/g of extract. The total content of flavonoids was 1.28 *μ*g of catechin equivalents per gram of extract. Likewise, the antioxidant capacity, determined by testing reducing power of Fe^3+^ was of 9.75 ± 0.17 *μ*g/mg of ascorbic acid equivalent.

### 3.3. Evaluation of the Gastroprotective Effect

#### 3.3.1. Effect on Ethanol-Induced Gastric Ulcers

**Figure 1:**
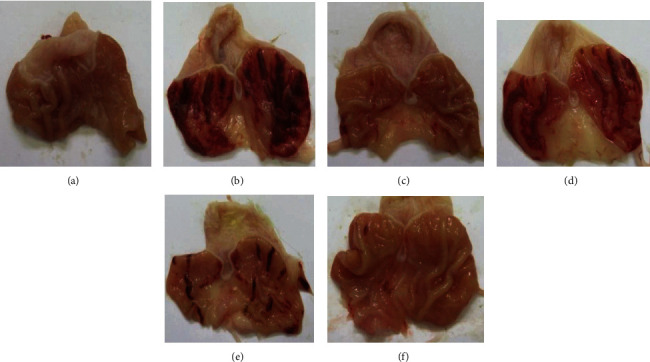
Photographs of rat stomachs with ethanol-induced lesions. (a) Negative control. (b) Positive control. (c) Omeprazole + ethanol. (d) *S. multiglandulosa* extract 100 mg/kg + ethanol. (e) *S. multiglandulosa* extract 250 mg/kg + ethanol. (f) *S. multiglandulosa* extract 500 mg/kg + ethanol.

**Figure 2:**
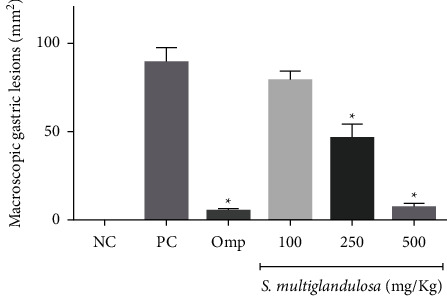
Effect of the hydroalcoholic extract of *S. multiglandulosa* on gastric lesions induced by absolute ethanol. Results are expressed as mean ± SD values for each group of 6 rats each. ^*∗*^*P* < 0.001 in relation to the ethanol group. NC: negative control; PC; positive control (ethanol).

Macroscopic analysis of the rat stomachs revealed an increased area of ulcerative or hemorrhagic lesions caused by the administration of absolute ethanol (89.44 ± 8.29 mm^2^). There was no statistical difference in mucus production between the control group (group I) and the ethanol group (group II). In the production of GS-NP if there was statistical significance between groups I and II (*P* < 0.001). The hydroalcoholic extract of *S. multiglandulosa* at doses of 250 and 500 mg/kg produced a significant decrease in the injured area, with a value of 46.28 ± 7.95 mm^2^ and 6.91 ± 2.48 mm^2^, respectively (*P* < 0.001). The lesion area observed in the group that received omeprazole was 4.83 ± 1.14 mm^2^ (protection equivalent to 94.60%), which does not differ statistically from the obtained result at the doses of 500 mg/kg, which had a protection of 92.27%. Figures 1 and 2.

**Figure 3:**
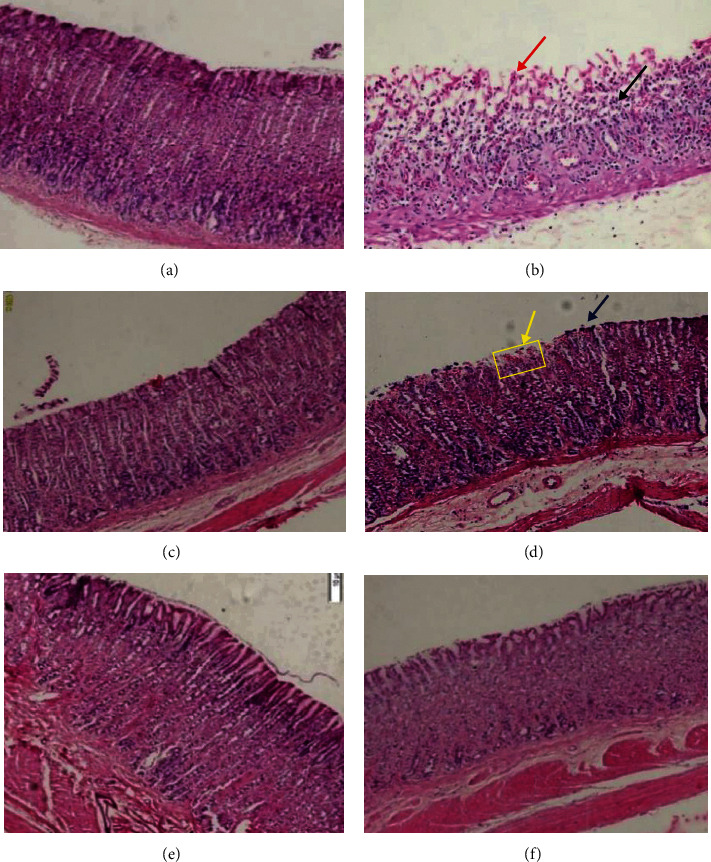
Photomicrographs of gastric mucosa of rats with lesions induced by absolute ethanol. (a) Negative control: normal tissue; (b) positive control; the red arrow indicates edema and chronic lymphocytic inflammatory infiltrate, the blue arrow indicates focal hemorrhage. (c) omeprazole + ethanol; normal tissue. (d) *S. multiglandulosa* extract 100 mg/kg + ethanol; The blue narrow indicates lymphoplasmacytic infiltrate, focal hemorrhage (yellow narrow). (e) *S. multiglandulosa* extract 250 mg/kg + ethanol; normal tissue. (f) *S. multiglandulosa* extract 500 mg/kg + ethanol; normal tissue.

Histopathological analysis of the gastric mucosa of rats showed no alterations in the normal control group (Figure 3(a)). In the group that received only absolute ethanol, edema, vascular congestion, focal hemorrhage, and chronic lymphocytic inflammatory infiltrate were observed (Figure 3(b)). No alterations were observed in the group treated with omeprazole (Figure 3(c)). In the groups treated with the hydroalcoholic extract of millhua, with the dose of 100 mg/kg mild lymphoplasmacytic inflammatory infiltrate was observed in some cases and hemorrhagic foci (Figure 3(d)), while at doses of 250 and 500 mg/kg no alterations were observed. in the gastric mucosa (Figures 3(e) and 3(f)).

In Table 2, the hydroalcoholic extract of *S. multiglandulosa* with doses of 250 and 500 mg/kg produced a significant increase in the production of mucus in the stomach of rats, with 500 mg/kg the value of 83.13 ± 13.09 mg/mL/g of tissue compared to 51.59 ± 8.60 mg/mL/g of tissue from the ethanol group (*P* < 0.05), this increase represented 61.14%. The production of nonprotein sulfhydryl groups (NP-GS) in the stomach also increased significantly with the three doses in a dose-dependent manner, reaching the value of 250.34 ± 21.16 *μ*g/g tissue with the 500 mg/kg dose compared to 113.82 ± 17.19 *μ*g/g tissue from the ethanol group (*P* < 0.05), this increase was 119.94%. Table 2.

## 4. Discussion

This study revealed that the most abundant secondary metabolites in the hydroalcoholic extract of *S. multiglandulosa* leaves were phenolic compounds, and flavonoids. In a study conducted in Africa, the presence of quinones in *S. multiglandulosa* leaves was also reported as the main abundant metabolite [20]. Other metabolites identified in another study from Ecuador were emodin; floribundone-1; torosanin-9′,10′-quinone; anhydrophlegmacin, 1,4 quinone moiety, kaempferol-3-O-galactorhamnoside; *α*-amyrin, *β*-sitosterol among other [21].

The total phenolic content found in the hydroalcoholic extract of *S. multiglandulosa* was slightly higher than those reported for blueberry (8.4 mg·GAE/g) and BlackBerry (7.40 mg·GAE/g) [22]. On the other hand, polyphenols are a group of more than 8,000 molecules that are only found in the plant kingdom, are produced as secondary metabolites and show remarkable antioxidant activity [23]. In this work, the presence of the high content of polyphenols is related to the antioxidant activity demonstrated in this investigation. Polyphenols have the property of scavenging free radicals due to their nonspecific actions, based on the common chemical characteristic that is the presence of the phenol group [24].

In the evaluation of the gastroprotective effect, several investigations of the family and isolated compounds of Senna genus have been reported in the literature. According to Hwang and Jeong [25] sennoside A and B administered at doses of 100 mg/kg had gastroprotective effect on ethanol-induced gastritis and indomethacin in male Sprague-Dawley rats and the main mechanism would be involved with the up-regulation of PGE2 prostaglandin and the inhibitions of *H*+/*K* + -ATPase activity. In the study of Karthikeyan and Gobianand [26], determined the antiulcer activity of *S. auriculata* leaves at different doses against pylorus ligation-induced gastric ulcer. The effective treatment was established at doses of 750 mg/kg of administration. In addition, some mechanism would be focused on the antioxidant activity, a decrease in gastric acid secretion, restoration of mucosal secretion and inhibiting the generation of lipid peroxidation. According to Guarize et al. [27] *Senna macranthera* leaves showed anti-inflammatory effect in an experimental model carrageenan-induced paw edema in rats with results similar like sodium diclofenac. Furthermore, it is important emphasize that the n-hexane extract could be involved with the laxative effect compared with other extracts.

The gastric mucosa maintains its physiological role through a balance between aggressive and protective factors. The overproductions of endogen aggressive agents result in ROS generation such as hydroxyl radicals, superoxide anions, and hydrogen peroxide, in addition a decrease of the defense antioxidant like catalase, glutathione, superoxide dismutase, etc. This imbalance is simulated with the use of ethanol in this experimental model to produce gastric mucosal erosion and ulcer [28]. In the present study, there was an increase in gastric mucus and nonprotein sulfhydryl groups, which includes glutathione, contributed in part to the gastroprotective effect of S*. multiglandulosa* extract, to allow the gastric mucosa to resist against the damage produced by absolute ethanol. This was probably due to the antioxidant effect of polyphenols, mainly flavonoids which were reported in this medicinal plant.

Endogenous sulfhydryl groups (SH) are related with the integrity of the gastric mucosal and support the aggressive attack of acid agents like ethanol and other chemicals inducer of gastritis. Furthermore, SH is adhered to the mucus layer creating a barrier against the proteolytic digestion. In this study the dose at 500 mg/Kg produces an increase of mucus and NP-SG (nonprotein sulfhydryl groups) being similar to omeprazole. Thus, it is likely that part of the gastroprotective mechanism of action would be related to the generation of mucus and NP-SG. It is known that these SH compounds form disulfide bridges with the mucus avoiding the solubility of this. Additionally, SH binds the free radicals leading to its antioxidant activity which would control the mucus production.

## 5. Conclusion

In conclusion, our results indicate that the oral administration of the hydroalcoholic extract from *S. multiglandulosa* leaves protected against ethanol-induced gastric ulcer in rats. The probable mechanism would be to the increase in gastric mucus secretion, the antioxidant activity and the increase of nonprotein sulfhydryl groups.

## Figures and Tables

**Table 1:** Phytochemical screening of the hydroalcoholic extract of *Senna multiglandulosa* leaves.

Reagent	Chemical group	Results
Molisch	Carbohydrates	+++
Ninhydrin	Amino acids	++
FeCl3	Phenolic compounds	++++
Gelatin	Tannins	+
Shinoda	Flavonoids	+++
AlC_l3_	Flavonoids	++
Liebermann–burchard	Steroids	+++
Dragendorff	Alkaloids	+++
Mayer	Alkaloids	++
Fehling	Reducing sugars	++
Rosenheim	Anthocyanins	+++
Foam test	Saponins	+++

(−) negative, (++) moderate, (+++) abundant, and (++++) very abundant.

**Table 2:** Effect of the hydroalcoholic extract of *S. multiglandulosa* on the production of mucus and nonprotein sulfhydryl groups (NP-SG) in rats.

Group	Mucus content		NP-SG	
	mg/mL/g tissue	% Increase	*μ*g/g tissue	% Increase
I: Negative control (physiological saline)	53.8 ± 11.67		244.28 ± 20.76	
II: Positive control (absolute ethanol)	51.59 ± 8.60		113.82 ± 17.19	
III: Omeprazole	102.43 ± 10.24^*∗*^	98.55	284.97 ± 14.81^*∗*^	150.37
IV: *S. multiglandulosa* 100 mg/Kg	55.93 ± 8.63	8.41	164.06 ± 15.79^*∗*^	44.14
V: *S. multiglandulosa* 250 mg/Kg	76.80 ± 10.07^*∗*^	48.87	220.76 ± 17.81^*∗*^	93.96
VI: *S. multiglandulosa* 500 mg/Kg	83.13 ± 13.09^*∗*^	61.14	250.34 ± 21.16^*∗*^	119.94

Values expressed as mean ± SD. ^*∗*^*P* < 0.05 compared to the ethanol group.

## Data Availability

The data used to support the findings of this study are included within the article.
